# *Pneumocystis* Pneumonia Presenting as an Enlarging Solitary Pulmonary Nodule

**DOI:** 10.1155/2016/1873237

**Published:** 2016-08-28

**Authors:** Krunal Bharat Patel, James Benjamin Gleason, Maria Julia Diacovo, Nydia Martinez-Galvez

**Affiliations:** ^1^Department of Pulmonary & Critical Care Medicine, Cleveland Clinic Florida, Weston, FL, USA; ^2^Department of Pathology, Cleveland Clinic Florida, Weston, FL, USA

## Abstract

*Pneumocystis* pneumonia is a life threatening infection that usually presents with diffuse bilateral ground-glass infiltrates in immunocompromised patients. We report a case of a single nodular granulomatous* Pneumocystis* pneumonia in a male with diffuse large B-cell lymphoma after R-CHOP therapy. He presented with symptoms of productive cough, dyspnea, and right-sided pleuritic chest pain that failed to resolve despite treatment with multiple antibiotics. Chest X-ray revealed right lower lobe atelectasis and CT of chest showed development of 2 cm nodular opacity with ground-glass opacities. Patient underwent bronchoscopy and biopsy that revealed granulomatous inflammation in a background of organizing pneumonia pattern with negative cultures. Respiratory symptoms resolved but the solitary nodular opacity increased in size prompting a surgical wedge resection which revealed granulomatous* Pneumocystis* pneumonia infection. This case is the third documented report of* Pneumocystis* pneumonia infection within a solitary pulmonary nodule in an individual with hematologic neoplasm. Although* Pneumocystis* pneumonia most commonly occurs in patients with HIV/acquired immunodeficiency syndrome and with diffuse infiltrates, the diagnosis should not be overlooked when only a solitary nodule is present.

## 1. Introduction

*Pneumocystis* pneumonia (PcP) is an opportunistic and potentially life threatening fungal infection that occurs in immunocompromised states. It is most commonly encountered in patients with HIV/AIDS and hematopoietic and solid malignancies and those receiving glucocorticoids and chemotherapeutic agents and other immunosuppressive agents [1]. Conventionally, it has been described as a bilateral, diffuse pulmonary disease having a histologic appearance of intra-alveolar eosinophilic foamy exudates containing cysts of* P. jirovecii* [2–4]. Granulomatous PcP accounts for approximately 5% of all PcP cases in AIDS patients, but the incidence in non-HIV patients is unknown due to a paucity of data [5]. A literature search revealed 17 previous cases of granulomatous PcP in patients with hematologic neoplasms [1, 6–9]. Of these 17 published cases, only two patients presented with a solitary pulmonary nodule and only two previous reports of granulomatous PcP have been published with large B-cell lymphoma [6, 7]. Awareness of a solid pulmonary nodule and granulomatous reaction is important as a single nodule could be seen as lymphoma involvement of the lung. In addition, it is important to realize that the diagnostic modality of bronchoalveolar lavage, which is typically done when diffuse infiltrates are present, may be of low yield if the organisms have not infiltrated in the alveolar lumen [7, 9]. Here we describe a case of diffuse large B-cell lymphoma complicated by granulomatous PcP presenting as an enlarging solitary pulmonary nodule.

## 2. Case Report

**Figure 1:**
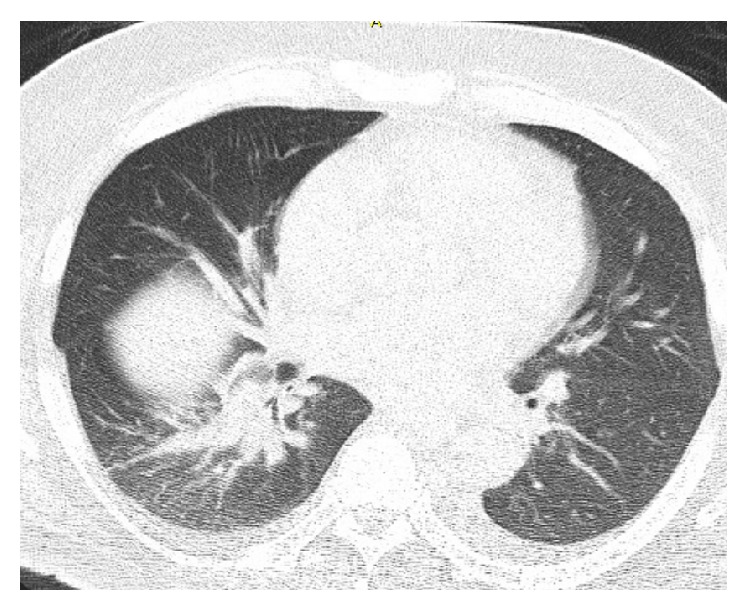
Axial image of CT of chest performed on August 17, 2013: pulmonary windows demonstrating atelectatic change or scarring in the right lower lobe.

A 61-year-old male was diagnosed with stage IIIB diffuse large B-cell lymphoma in August 2013. He underwent treatment with R-CHOP (rituximab, cyclophosphamide, hydroxydaunorubicin, oncovin, and prednisolone) chemotherapy. Two weeks after completing a total of six cycles of CHOP and eight cycles of rituximab he presented to our office for evaluation of dyspnea on exertion, cough with clear sputum production, night sweats, and right-sided pleuritic chest pain. Review of his chart showed a previous Computed Tomography (CT) imaging of the chest with atelectatic change or scarring within the right lower lobe (Figure 1).

**Figure 2:**
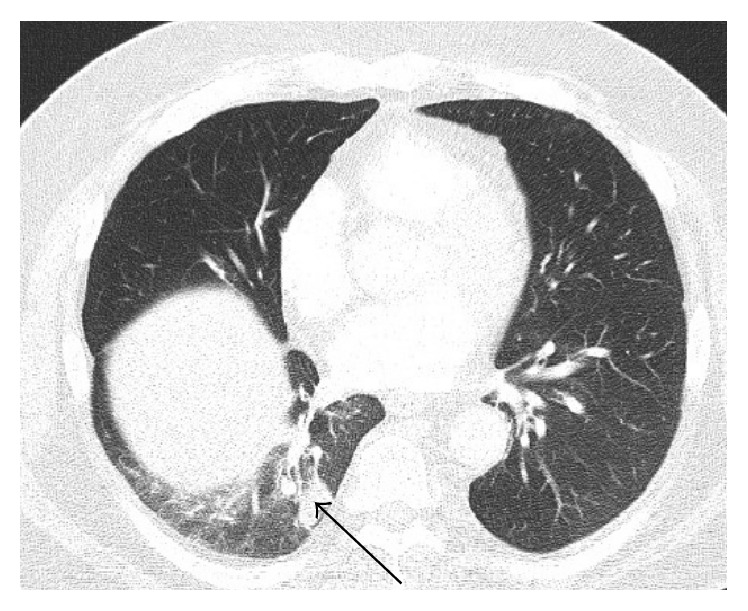
Axial image of CT of chest performed on January 31, 2014: pulmonary windows demonstrating a 2.3 cm nodular opacity in the right lower lobe (arrow) with minimal subsegmental atelectatic changes and ground-glass opacity.

On this visit, chest X-ray showed bibasilar infiltrates/atelectasis and CT of chest showed 2.3 cm nodular opacity in the right lower lobe with minimal subsegmental atelectatic changes and ground-glass opacity (Figure 2). Positron Emission Tomography-Computed Tomography (PET-CT) imaging showed a mildly 18F-fluorodeoxyglucose avid peripheral right lung mass coincident with the previously noted nodular opacity.

**Figure 3:**
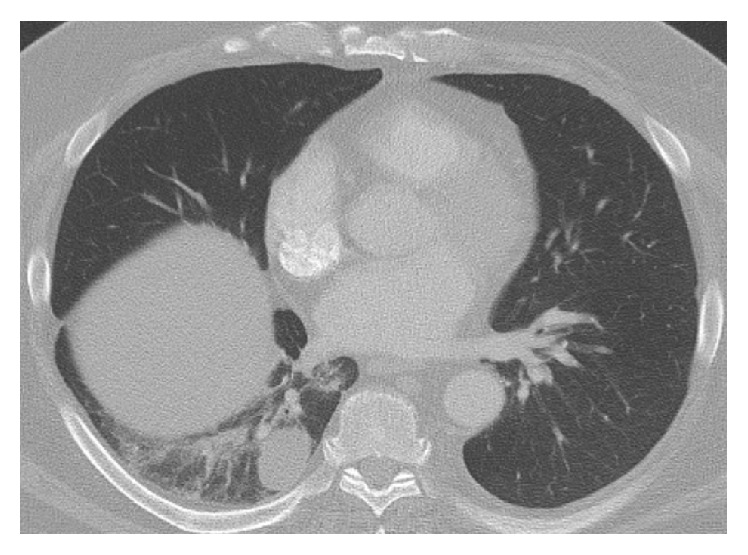
Axial image of CT of chest performed on April 15, 2015; pulmonary windows demonstrate an enlarging 3.6 cm nodular opacity in the right lower lobe with minimal subsegmental atelectatic changes and ground-glass opacity.

**Figure 4:**
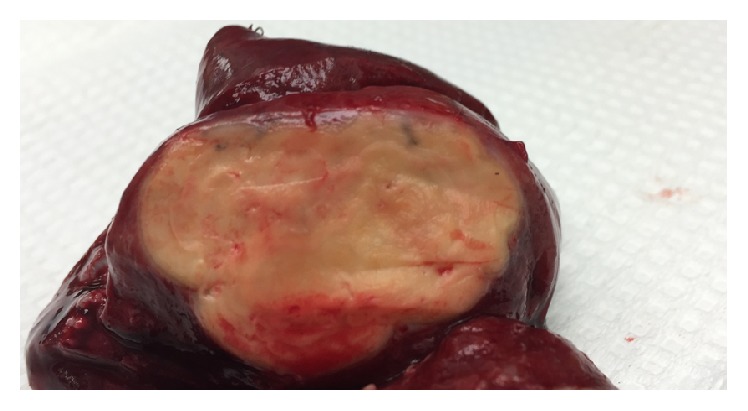
Right lower lobe wedge resection gross surgical specimen: the solid nodule measuring 3.6 cm demonstrating smooth lobulated contours and a yellow-tan resilient surface.

**Figure 5:**
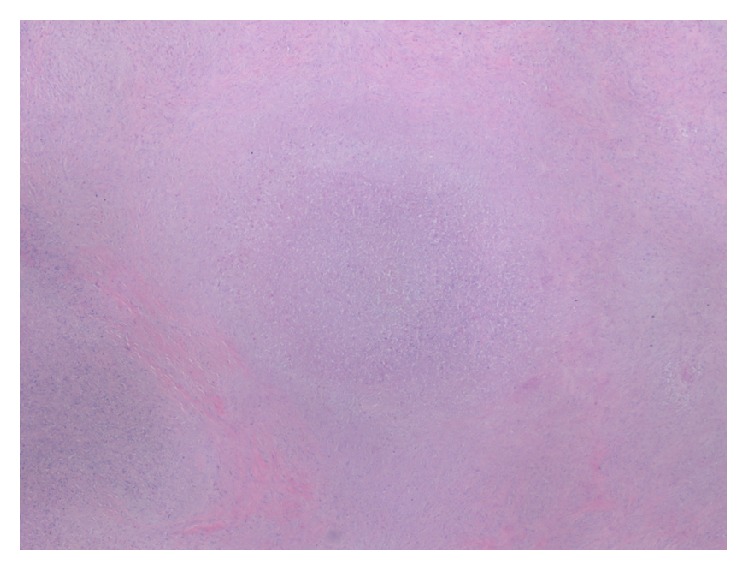
Microscopy 4x magnification: Hematoxylin and Eosin stain demonstrating confluent necrotizing granulomas with peripheral bands of sclerosis.

**Figure 6:**
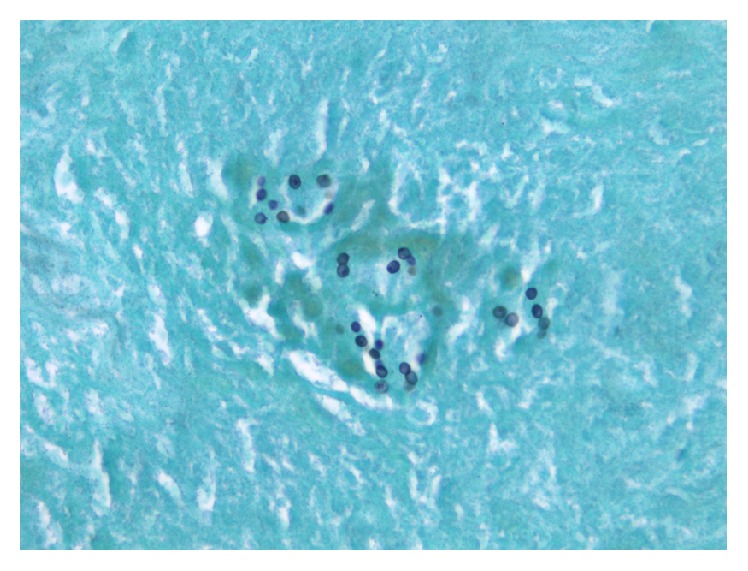
Microscopy 800x magnification: Gomori methenamine silver stain demonstrating classic* Pneumocystis jirovecii* cysts (cup-shaped with central dark zone, 5–7 *μ*m) embedded within granuloma necrosis.

Bronchoscopy with bronchoalveolar lavage (BAL) was performed and was negative for infectious (AFB, fungal, and bacterial) and malignant causes.* Pneumocystis jirovecii* was not identified in the fungal smear and gram-stain or subsequent cultures. Because of his atypical presentation* Pneumocystis jirovecii* PCR assay was not performed on the lavage sediment. He was referred for CT guided core needle biopsy and obtained specimens showed granulomatous inflammation in a background of organizing pneumonia pattern. A silver stain was not performed on the core due to limited available sample. The core sample was felt to be nondiagnostic and surgical lung biopsy was recommended but the patient was lost to follow-up. When he returned 1 year later, he was asymptomatic but follow-up CT imaging of the chest revealed an enlarging right lower lobe mass (Figure 3). He underwent video-assisted thoracoscopic surgery (VATS) with right lower lobe wedge biopsy (Figure 4) which disclosed necrotizing granulomatous inflammation with background pulmonary parenchyma with organizing pneumonia and mixed inflammation (Figure 5). Silver stain was performed on the surgical specimens and identified* P. jirovecii* cysts associated with necrosis within the granuloma (Figure 6). He was started on oral trimethoprim/sulfamethoxazole 160 mg/800 mg; however due to intolerance his regimen was changed to atovaquone 750 mg by mouth twice daily for 12 days. He responded well to this treatment and follow-up CT imaging of the chest revealed no further progression of disease.

## 3. Discussion

PcP is commonly described with the radiologic appearance of diffuse bilateral alveolar and interstitial infiltrates with perihilar distribution along with pathologic findings of foamy intra-alveolar eosinophilic exudates containing the PcP organisms [2]. Most patients present with the typical symptoms of cough and dyspnea in the setting of immunodeficiency. A granulomatous response has been previously described as an unusual histologic finding and primarily occurs in HIV/AIDS patients.

The pathogenesis of granulomatous reaction in PcP is related to host factors. These include active malignancy, recent corticosteroid use, Immune Reconstitution Inflammatory Syndrome, and prophylaxis with pentamidine rather than PcP genotypes [4]. Patients such as ours, with R-CHOP for lymphoma treatment, are at increased risk of developing PcP and in many cases are overlooked.

Diagnosis of conventional* Pneumocystis* pneumonia has traditionally involved bronchoalveolar lavage. However, a literature review suggests that bronchoscopy is considered to have a very low diagnostic yield, if any, to detect* P. jirovecii* granulomas [9]. Despite this reports of granulomatous PcP being diagnosed by PCR assay of bronchoscopically obtained BAL sediment have been stated [10]. Despite the definitive identification of* P. jirovecii* granulomas in this patient other possible etiologies for enlarging pulmonary nodules after completion of antineoplastic therapy need to be ruled out. These possibilities include large B-cell lymphoma recurrence (in this case), primary pulmonary malignancy, residua of infection simultaneous to therapy, typical pulmonary bacterial infections, and even endemic mycoses such as histoplasmosis, blastomycosis, and coccidiomycosis [11]. In our case surgical lung biopsy is necessary to rule out these other possibilities and obtain a definitive diagnosis.

To date, our case is the third report of a* Pneumocystis jirovecii* infection within a solitary pulmonary nodule as well as the third report of granulomatous* Pneumocystis jirovecii* in an individual with diffuse large B-cell lymphoma. Additionally, we describe the only case which demonstrated an enlargement of the nodule over a span of 1 year and resolution of symptoms with treatment.

In conclusion, this case demonstrates that granulomatous PcP infections may exhibit rare radiologic manifestations including nodular opacities and affected patients may have subacute or even chronic symptoms. Consequently, granulomatous PcP should be considered in the differential diagnosis of solitary pulmonary nodules in all immunocompromised patients beyond the classically described AIDS affected.
